# The Experience of Adults Bereaved by the Suicide of a Close Elderly Relative: A Qualitative Pilot Study

**DOI:** 10.3389/fpsyg.2020.538678

**Published:** 2020-09-14

**Authors:** Gabrielle Michaud-Dumont, Sylvie Lapierre, Charles Viau-Quesnel

**Affiliations:** ^1^Laboratoire interdisciplinaire de recherche en gérontologie (LIREG), Université du Québec à Trois-Rivières, Trois-Rivières, QC, Canada; ^2^Centre for Research and Intervention on Suicide, Ethical Issues and End-of-life Practices (CRISE), Université du Québec à Montréal, Montréal, QC, Canada

**Keywords:** suicide, grief, bereavement, older adults, family, close relatives

## Abstract

Suicide in older persons is a serious issue in many countries. The act of intentionally causing one’s own death is often associated with lack of social support, thwarted belongingness, or chronic interpersonal difficulties. Therefore, suicide has a significant interpersonal dimension that can influence those left behind. However, studies that have investigated the impact of older adults’ suicide on their family are scarce. The objective of this pilot study was to assess the feasibility of a qualitative research on the psychosocial experience of adults bereaved by the suicide of an elderly relative. This research could recruit three participants (daughter, grand-son, and grand-niece) who had lost to suicide a close family member aged between 75 and 90. The analysis of the content of the semi-structured interviews revealed seven main themes: (1) finding an explanation to the suicide, (2) give meaning to the loss, (3) the emotional processes of mourning, (4) the repercussions of the suicide on the individual and the family, (5) looking for support, (6) the taboo and secrecy of suicide, and (7) perceptions of aging and the end of life. To explain the suicide of their loved one, the bereaved mentioned various factors related to aging, such as loss of autonomy, illness, and fear of placement. Although the older relative was perceived to be approaching death because of his/her age, the suicide was still unexpected and shocking and led to various emotions (shock, anger, and guilt) and to family conflicts. Suicide remains a taboo subject, but the newly legalized medical assistance in dying is seen as a potential solution to suffering in old age. Further investigation is clearly needed on this topic and this pilot study indicates that the main difficulties will lie in the choice of selection criteria for participants and in the recruitment process.

## Introduction

Suicide is a public health problem among older adults (Richard-Devantoy et al., 2013; World Health Organization, 2014). According to the World Health Organization (2020), the percentage of the world’s population over the age of 60 will double from 2000 to 2050, from around 11 to 22%. Although the suicide rate for people aged 65 years and older has fallen in the province of Québec (Canada) since the 1980s, the total number of deaths by suicide has actually risen by 40% due to the growing number of individuals in this age group (Association québécoise de prévention du suicide, 2014). In 2017, the suicide rate for older adults was 11.3; however, it was five times higher for men (20.6) than women (3.7; Levesque et al., 2020).

Suicide is a serious issue that takes place in a context of interpersonal relationships between the person who died by suicide, family members, and close acquaintances. Any research concerning suicide in the older age group would be incomplete without accounting for the perspective of the bereaved (Andriessen and Krysinska, 2012). Accordingly, the issue of late-life suicide must be addressed in order to prevent the negative repercussions on family and friends. Previously, it has been estimated that for each completed suicide, between 6 and 10 persons were profoundly affected by the death (Cerel et al., 2008). New data, from a random-digit dial survey (*N* = 1736, where veterans were deliberately oversampled), lead to an estimate of 135 adults exposed per suicide death (Cerel et al., 2019). In addition, because suicide by an older person remains a taboo subject, and because it is considered normal for older people to die, the bereaved find it more difficult to acknowledge their grief (Figueiredo et al., 2012). Furthermore, suicide in older adults may be viewed as a rational act when the person is suffering from a chronic debilitating disease, as well as a means of controlling the timing of one’s death while escaping the prospect of burdening others. For these reasons, many may view suicide in old age as an “acceptable” solution to a predicament (Cerel et al., 2008). Yet, few studies have investigated the impact of the suicide of an older person on their close adult relatives (Cerel et al., 2008). It is possible that these bereaved adults would have specific experiences and needs that differ from those of other bereaved individuals (Harwood et al., 2002).

To address this issue, it is useful to adopt a theoretical approach based on suicide bereavement and postvention (Andriessen et al., 2017). The literature on suicide has shown that this type of death seems to expose the bereaved to a complex grieving process and that postvention (intervention support for the bereaved) could be an important strategy for suicide prevention since survivors of suicide loss seem to have a higher risk of suicidal behavior (Andriessen, 2009). Bereavement after a suicide comprises various prominent and intense thematic issues (Jordan, 2008). After the shock that usually accompanies the event, survivors of the loss must cope with mixed feelings, unanswered questions, family relational disturbance, and social stigma. In addition, they frequently report feelings of guilt, shame, and social isolation, not to mention negative consequences for their social, physical, and mental well-being (Jordan, 2008; Tal Young et al., 2012; Pitman et al., 2014). Suicide has an undeniable psychosocial impact on the survivors.

Nevertheless, whereas the literature is abundant on individuals who have lost a close relative or friend to suicide, the survivors, as well as the deceased, are usually aged under 65. Few studies have involved adults whose older relative intentionally ended their life (Kjølseth et al., 2009). Among the existing research, Harwood et al. (2002) showed that relatives (children, spouses, and sibling) and friends of older adults who died by suicide experienced more stigma, shame, and sense of rejection than relatives of older adults who died from natural causes. One qualitative study, done in Brazil by Figueiredo et al. (2012), showed that family members of older people who died by suicide reported social stigma and prejudice, social isolation, feelings of guilt, rage, and anguish, and for some, irreverence. They tried to distance themselves from the location of the suicide (the home) since it had become a threatening environment for them. Survivors also mentioned that they had not believed that their older relative would end their life even when the latter explicitly said they would.

With the scarcity of studies on the topic, it remains unclear whether adults who faced the suicide of an older relative suffer similar or different experiences and consequences, as those reported in studies with other groups of bereaved individuals, and if they have a need for particular support. It seems necessary to fill this gap in the literature. However, it also appears relevant to include an internal pilot in the design of our main study in order to see if adjustments are necessary to the methodology since it might be difficult to recruit participants due to the stigma and feelings of shame associated with suicide (Cerel et al., 2008; Janghorban et al., 2014). Moreover, since the aim of the main study is to make an insightful analysis of the experience of the bereaved, it is necessary to verify the suitability of the instrument to increase confidence in the trustworthiness of the data that may be obtained (Malmqvist et al., 2019). In addition, the pilot study could provide a ground for self-assessment of the main researcher’s preparation and capacity to practice a qualitative inquiry, for improving skills in conducting an in-depth interview and seizing opportunities for probing emerging topics during the interview process; all of which would enhance the credibility of the research (Nunes et al., 2010; Padgett, 2016). In fact, a well-planned and thoroughly conducted internal pilot study would ensure higher research quality when a depth of understanding is sought (Malmqvist et al., 2019). Thus, the objective was to have a preliminary overview of the psychosocial experience of adults bereaved by the suicide of an elderly relative and to assess the feasibility of a qualitative research on this topic in order to improve the efficiency and quality of the forthcoming main study.

## Methods

### Study Design

Given the lack of studies on individuals bereaved by the suicide of an older family member (Cerel et al., 2008; Figueiredo et al., 2012), a qualitative approach was deemed appropriate to examine the psychosocial experience of the survivors following this event (Hjelmeland and Knizek, 2010). Therefore, we used a semi-structured interview to question the participants about their experience at the time of the loss. All potential participants that were contacted for the study received information about the suicide prevention center of their region, which offers services to the bereaved, or about the psychological clinic of the university, in case they needed it.

A number of selection criteria were established to ensure that potential participants were not overly vulnerable. First, to be included in the study, they had to be older than 18 years. Second, they had to have lost to suicide a parent or a grandparent aged 65 years and older between 2 and 20 years ago. The 2-year minimum was expected to give participants sufficient time since the death to step back from the event and to be able to reflect on their experience, while the 20-year maximum was chosen according to the number of years since the suicide, observed by local prevention centers in members of support groups for survivors. Third, they could neither present a diagnosis of depression or other mental health disorders nor should they report suicidal ideation, as verified by the PHQ-9, described below.

### Ethical Consideration

This research was approved by the Human Research Ethics Board of the University of Québec in Trois-Rivières^1^ and different strategies were planned to minimize risks to participants considering that the area of suicide-bereavement is highly sensitive and people who have been impacted are extremely vulnerable. The major risk associated with this study refers to the reactivation of painful memories and the discomfort caused by certain questions. Although some bereaved people may find the interview difficult and even upsetting, it should be noted that a few studies have concluded that participants usually assess this type of interview positively and find it useful (Cooper, 1999; Dyregrov et al., 2011; Andriessen et al., 2018). A safety plan has also been formulated to protect participants. The interviewer paid special attention to signs of complicated grief, excessive stress, or suicidal ideations during the meeting and asked about their current emotional state at the end of the interview. After the interview, they were referred to psychosocial resources who could support them in their mourning, such as the psychological clinic of the university, and were given the 24-h contact number of the Centre for Suicide Prevention.

### Procedure

Participants were recruited through advertisements posted in public areas of the university and in various coffee shops around the city of Trois-Rivières. The local university has over 15,800 students or employees aged between 20 and 65 years. The advertisements were also posted on social networking websites (e.g., Facebook) and widely published online by suicide prevention centers. People who showed interest in the study were scheduled for an initial telephone interview. The purpose of this interview was to explain the research objective, the potential benefits and risks of participating, and the duration and nature of their participation. It also led to the verification of the inclusion and exclusion criteria. It should be noted that four other participants contacted the research coordinator, but they were excluded because their relative was between 50 and 65 years old. They were referred to another study investigating the experience of people bereaved after the suicide of a “babyboomer”.

The French translation of the Patient Health Questionnaire (PHQ-9) was used to screen for depression and suicidal ideations (Kroenke et al., 2001). It contains nine questions and takes about 5 min to complete. It has 77% sensitivity and 85% specificity (Institut national d’excellence en santé et en services sociaux, 2015). Participants who scored higher than 4 (indicating at least minimal depression) or who reported suicidal ideations on item 9 would be excluded from the study and referred to a mental health resource, as desired. No potential participant was excluded. Consequently, a date was set for a semi-structured face-to-face individual interview. The interviews took place at the Interdisciplinary Laboratory for Research in Gerontology (LIREG) at the University of Quebec in Trois-Rivières with the principal investigator (GMD) and the participant only. The principal investigator of this study is currently completing a doctorate degree (PhD) in clinical psychology. She also took a 45-h university course on qualitative research, in addition to various suicide prevention training.

Prior to being interviewed, the participants signed a voluntary informed consent form. No participant received any monetary compensation for participating in the study. The interview covered various topics with 13 questions intended to incite participants to recount their experience and express their thoughts and emotions concerning the suicide of their older relative (see Appendix 1). It was developed by the principal investigator on the basis of existing research on people bereaved by suicide. When they expressed intense emotions, the researcher showed empathy and slowed the pace of the interview to allow them the time needed to communicate. Field notes were taken by the researcher throughout the interview in order to identify the main ideas and to further question the participant on his/her experience. The interviews lasted from 43 to 105 min (see Table 1). Each participant was assigned a numerical code to ensure anonymity.

**Table 1:** Demographic characteristics of the interviewees and the deceased.

Interviewee	Gender	Age	Deceased’s relationship to the interviewee	Deceased’s age at suicide	Time since the suicide (years)	Length of interview (minutes)
A	Male	20–25	Grandfather	80–85	3	50
B	Female	30–35	Great aunt	75–80	15	44
C	Female	60–65	Father	85–90	2	105

### Participants

Participants were recruited from the general population over a 15-month period. Initially, we intended to recruit children and grandchildren only, but since it was difficult to find adults who were willing to share their experience, we also accepted a participant who had lost her grandaunt with whom she was emotionally close. The sample of the current study includes three adults who lost a close relative whose age at the time of the suicide varied from 75 to 90 years. We had planned to recruit at least 12 participants, as recommended by certain studies (e.g., Isaacs, 2014) on qualitative research methodology, but we could only recruit three. The bereaved participants consisted of a man between 20 and 25 years of age, as well as two women aged between 30–35 and 60–65, respectively. These age ranges are used to ensure anonymity considering the small sample. The time since the suicide ranged between 2 and 15 years. Table 1 presents some information on the nature of the relationship between the bereaved and the deceased and their demographic characteristics. Interviews were held from June to November 2019.

### Data Analysis

An inductive method was chosen for the data analysis as current research on adults bereaved by the suicide of an older person remains scarce (Thomas, 2006). This conventional method has the advantage of producing reliable knowledge through in-depth examination of a limited number of cases (Miles et al., 2013). First, the interviews were audio-recorded and fully transcribed. The verbatims were not returned to the participants for comments or corrections. Then, the first author performed a thematic analysis of the interviews’ content following a step-by-step guide suggested by Braun and Clarke (2006). During the analysis, the investigator attempted to preserve the original meaning by making a full and accurate representation of the participants’ statements, including pauses and tone. Repeated and immersive readings of the verbatim transcriptions led to the identification of recurring themes, which were coded by the first author using the qualitative software NVivo 12, as it facilitated preliminary thoughts to emerge across cases and develop linkages between categories and initial themes (Bengtsson, 2016). Therefore, analysis first began with assigning broad codes to the data, followed by building a second cycle of codes on the initial broad codes, and grouping them into meaningful categories or themes (Kalpokaite and Radivojevic, 2019). The themes were then discussed and refined with another researcher (SL) to establish interjudge agreement. The aim was to ensure that the categories accurately represented the participants’ experiences. Thus, based on multiple discussions, authors reached a high degree of consensus.

## Results

The analysis of the content of the semi-structured interviews revealed seven main themes: (1) finding an explanation to the suicide, (2) give meaning to the loss, (3) the emotional processes of mourning, (4) the repercussions of the suicide on the individual and the family, (5) looking for support, (6) the taboo and secrecy of suicide, and (7) perceptions of aging and the end of life.

### Theme 1: Finding an Explanation to the Suicide

Results indicated that the bereaved relatives tried to understand and justify the suicide by seeking various explanations for the act, all of which were associated with aging, physical illness, cognitive decline, and fear of placement in a nursing home. All three participants recounted that, prior to the suicide, their relative suffered from physical or neurological conditions. Furthermore, two of them had been diagnosed with Alzheimer’s disease a few months before they ended their life. Participant A specified that his grandfather had often voiced his fears about Alzheimer’s disease. *He had seen what Alzheimer’s was like, because there was a nursing home that he visited a lot. He knew about Alzheimer’s, and he was, sort of … not vain, but proud. He did not want to go through the Alzheimer stages, the decline. He knew everything that would happen, and it was out of the question for him to take that route* (Interviewee A, grandfather).

In addition, some participants believed that the way their relative learned about the diagnosis of Alzheimer disease was problematic and contributed to the exacerbation of the shock of the news.

*It was the start of Alzheimer’s. But what happened for the drugs (is that), the pharmacist and the nurse did not say it was for Alzheimer’s, and that it was really for memory problems. What really shocked him was when they called him for a renewal, and they said, “then for your Alzheimer’s drugs.” He did, “What? I do not have Alzheimer’s.” He was downright … As if it was a plot against him and everyone wanted to hide that it was Alzheimer’s, so it was a shock for him […] It’s sure there might have been something that should have been done. As much from professionals, as much as perhaps from us too. At the time, we did not know* (Interviewee A, grandfather).

Persistent physical pain, often listed among predisposing factors for suicide in older adults, also constitutes a common explanation mentioned by the participants (Harwood et al., 2006). *So, the next afternoon, a doctor came because he had gotten to a point that … He wasn’t eating, he was confused. In the end, he had sinusitis. […] It seemed like that marked the beginning of the end … (Interviewer: This was what trigged it, you think?). Yes. Then antibiotics … And from then on, his questions, they never stopped. We saw that … even in those days, and for the rest of his life, he was always in some kind of pain* (Interviewee C, father).

Transfer to a nursing home is a crucial moment in the life of an elderly person. However, the adaptation to this transition largely depends on the meaning attributed to this event. Two of the participants described the inability of their loved one to accept a placement in a long-term care facility, whether it was decided or a possibility.

*I think he hid his symptoms well. I think that he knew more about the stage he had reached in his illness than he let on. Or more accurately, being proud, he did not want to show it. “Oh no, I’m still able to live at home.” … He knew that if he said: “Hold on, there’s a lot of things going on in my head,” … He knew that he was going to be placed* (Interviewee A, grandfather).

*At the same time, we were very aware, well, … or rather we were sort of aware at the time, that she was sick. As for her, she had always told my father, “Me, I’ll never end up in a nursing home or a hospital, or whatever … If I get sick, I’m going to die in my own home”* (Interviewee B, grandaunt).

Although illness was the main reason mentioned to explain their relative’s suicide, participants also stated various losses (e.g., driving license, freedom) or the development of conditions, like dependency, that had major consequences on their relative’s quality of life and dignity. Everything that had given value to their lives had been lost. Losing a driver’s license can have serious consequences since its possession is intimately linked with older people’s identity and feelings of independence, agency, self-worth, and autonomy (Whitehead et al., 2006). Their older relative’s increasing feelings of powerlessness emerged through the descriptions of these disappointments and losses.

*So, she would say: “Nobody’s going to wipe me, that’s for sure.” And in the same way, “Nobody’s going to take care of me”* (Interviewee B, grandaunt).

*It was the Alzheimer’s that led to the suicide, it’s clear. Because he was still … he was still active. Another thing that happened, also, was the loss of his driving license, not long before. Because that’s what comes with it. He was very autonomous, my grandfather. He loved going out for a drive. He loved having his freedom, doing his things, going grocery shopping, and his little routine. […] So, it’s clear that this made him feel more isolated, you understand* (Interviewee A, grandfather).

In addition to the awareness of the deficits, the threat of placement, and the refusal to mourn the loss of one’s previous abilities, the feeling of being trapped was an element that seems important in the participants’ perception of the suicide. *I think that he was deteriorating and that he did not see any way out* (Interviewee C, father).

According to the participants, these older adults wanted to maintain control over their lives and solve their problems in their own way.

*The driving license, the fact that there was a place in a nursing home waiting for him, all that. That’s clear … Then, the fact that he had not already been told that he had Alzheimer’s. All that, the melting pot. I think that, in hindsight, me, I’d say, … He wanted to take care of it in his own way, before he lost his autonomy completely and became dependent. I think that it’s clear that he would not have wanted that! He would not want to be a burden. That would be out of the question* (Interviewee A, grandfather).

*Because sometimes we tell ourselves, yes, there’s always a way to make things better, and to ask for help, but sometimes, you may be fed up. So, it depends on the situation. For her, I think that she could have (ask for help), but at the same time, if that was always her mindset, “Me, I’ll never die in a hospital, and I do not need to be taken care of,” well, … she had decided that she wasn’t going into a hospital, and she wasn’t going to be taken care of* (Interviewee B, grandaunt).

Finally, some participants attributed the suicide to attitudes or behaviors believed to be held by the older generation. For example, participant B describes a lack of flexibility or a low level of “openness to experience” that characterize certain personality traits associated with suicide among seniors (Duberstein, 1995). The lack of openness to new experience is characterized by short-term concrete life goals, behavior that is not conducive to change, the absence or few interests, and a limited range of emotions.

*People aging as a couple live for their couple and there is nothing else. But at the same time, these people did not know what volunteering was. These people have known: we work and when we stop working… Well back in time, they would die when they stopped working. Today they do not die, but they do not know what to do. They were not brought up to that either and they were not brought up to talk about what they were going through* (Interviewee B, grandaunt).

### Theme 2: Give Meaning to the Loss

After looking for explanations for the suicide (see first theme), participants questioned their own visions of life and some took concrete action in order to reconstruct their views and get through the mourning process. For example, participant A described how the event had pushed him to study gerontology so he could increase his knowledge about mental illness and make sense of this loss in his life. He also found a job with older adults thereafter in order to get involved with this population and help them.

*I’m the only one in my family who said I’m going into gerontology. I want to understand more about what happened. So, with these courses, I have a better understanding of mental illness. It’s hard to understand something when you do not have the knowledge, you do not know the theories about it. It has helped me a lot in personal terms too […] I’ll tell you, that helped me get through my mourning […] I sort of experienced another vision of life. You could say that I was looking for something: a meaning. It made me question things. It brought out a lot of things that I had inside me. You know, philosophize about life and all that. Become more aware of what life is* (Interviewee A, grandfather).

The suicide also disturbed their convictions about the deceased. To make sense of the loss, the participants clung to diagnoses given by professionals. *Very narcissistic, so one could also imagine that feeling himself decline, for someone who’s … that must be very hard to live with, even for someone who is not narcissistic. So, one could imagine that … (Interviewer: This put additional stress). You would know better than me. But that’s my feeling. For me, that’s how I understand it. That’s how I made sense of it all* (Interviewee C, father).

All the participants were unanimous in their belief that understanding the suicide and their relative’s perspective on life helped them to make sense of it. Meaning reconstruction or the ability to change one’s view of a situation had a positive influence on their mourning process and on accepting the loss.

*For me, that’s how I overcame it, for sure. I wanted to understand. Knowledge about what really drove it [the suicide], and what had really made it happen. By working with older people, too, by witnessing their reality, by seeing other situations* (Interviewee A, grandfather).

*Me, I was less emotional, because I found that I could understand her reasoning. Put it this way: me, I do not want to die, or be sick, or suffer needlessly. So, I understand what she did* (Interviewee B, grandaunt).

#### Minor Theme: Desire to Help

Finding something positive in a negative experience helps to derive meaning from a loss and buffer against the psychological damage of such events (Sutin et al., 2010). Helping others is a way to turn a negative situation into something positive. Altruism seemed to be the central motivation among the bereaved to participate in a study on this topic. Indeed, they hoped that with their contribution they would help prevent suicide and help advance research and services for survivors (Dyregrov et al., 2011). It might also be a way to give meaning to their loss.

*Well it’s interesting [this study]. I liked it. I think it allowed to take a step back. We took it that way at the time, but there what we would like today? And then, yes finally, you realize that I would have liked to know her* more (Interviewee B, grandaunt).

*It’s good that we talk about it, it’s good that there are studies like that that are being done, I find it great, that’s why I signed up. When I saw it [the poster], I said to myself, I’m going to participate. I said to myself, I’m going to give a hand* (Interviewee A, grandfather).

### Theme 3: The Emotional Processes of Mourning

The emotions experienced by the participants were very intense at the beginning of the mourning process. On the day of the suicide, all the participants reported shock when they heard the news as well as great sadness. Although they might have expected their relative to die eventually, as a result of their condition and their advanced age, the suicide was experienced as a brutal and unexpected event. *We were not expecting it … yes, he was sick, but it had not yet gotten to the point that death was imminent or obvious. It was more brutal (Interviewer: You thought there was more time?). Yes […] Because never in my life would I have said that my grandfather was going to get Alzheimer’s, and that he was going to commit suicide, that’s for sure. Never, never, never, would the thought have occurred to me* (Interviewee A, grandfather).

All the participants said that they had forgotten some of what happened on the day of the suicide. Participants A and C, who had gone to the scene as soon as they received the news, described feelings of intense shock that seemed to plunge them into a black hole. *For the rest of the day, after the shock had settled, we talked, but to be honest, I do not remember what we said at all. The shock was a little too much* (Interviewee A, grandfather)*. That evening, the coroner came, but my mind was a blank. I do not know if anyone was with my mother when I got in touch with her (Interviewer: You do not remember?). A total black hole* (Interviewee C, father).

Two of the participants reported anger against the deceased. One of them associated this anger with her frustration at not being informed about the suffering of her relative, and therefore not having the opportunity to help her. However, this anger appeared to be attenuated by her awareness of the difficulty of passing judgment on other people’s lives. *Just tell us that you are suffering. At least we can try, if only to sympathize, help with the chores. I do not know. I tell myself, we lived far from each other, but I mean … maybe there was something we could have done all the same, but we did not know, because people do not tell us … Me, I say, you cannot blame someone … they have their reasons for doing it. It was too much for her, you do not know what’s going on in her mind, you cannot judge her* (Interviewee B, grandaunt). Another participant also expressed anger, particularly since she was very involved in caregiving for her older parent and had spent a lot of time meeting his needs. She associated her resentment with her perception of suicide as a selfish gesture (or lack of consideration for others) and to the tangible consequences for the people left behind. *He had no business doing that, leaving my mother all alone, she who took care of him for his whole life. And who left it all up to me. He left it all up to me […] I think that it’s selfish. I think that he realized that he was deteriorating, and he did not want to deal with the problems* (Interviewee C, father).

The participants also said they felt guilty, but this feeling was moderated by whether or not they felt they had done everything in their power at the time. For instance, participant A expressed remorse that he had not been there enough for his grandfather. However, he said that his mother felt even more guilty because she blamed herself for having missed the signs of suicide. In contrast, participant C refused to feel guilty because she believed that she had given everything she could to her father when he was alive and, therefore, had nothing to regret. *Guilty, no. I think that I gave him everything that I could. And even if I had understood what he might have intended, he acted the next day* (Interviewee C, father).

In association with the guilt, we noted regrets and the need to formulate diverse scenarios that might have led to a different outcome. These hypothetical scenarios began with: “If I could…” or “I should have …” or “If the situation had been…” *[My mom] was saying: “I could have, I should have been there, or if I had been there, if I had seen the rope, if I had realized,” … Thinking over everything that was said, going over all their conversations* (Interviewee A, grandfather). In participant C’s case, geographic distance led her to create a scenario in which she would have lived close by and might have noticed some signs of suicidal ideation. *I mean, we did not know what condition she was in. She would have been nearby, I would have seen her every week. I would have seen it eventually, I do not know, that she had lost weight, or that she was exhausted, or that she seemed in pain* (Interviewee B, grandaunt).

### Theme 4: Repercussions of the Suicide on the Individual and the Family

Suicide affects the personal lives of those left behind. Mourning after a suicide can become a profoundly isolating experience, one that may have a significant and quite deleterious impact on the survivor’s relationships with family and friends (Jordan, 2008). In addition to the sorrow that is common after all losses, two suicide survivors showed high levels of distress in several domains of their functioning. *I had the impression that I vegetated for months. […] I was very, very rattled by the whole thing. […] I never felt like going to my Tai Chi class. I did not feel like it. And I could not concentrate. There were even times when I had trouble reading. […] I stopped eating. I’ve gained back two pounds, but I lost 20 after my father died. My appetite is not very good. […] I was assessed by a psychiatrist, who told me that it was impossible for me to go back to work* (Interviewee C, father).

They clearly stated that their priority was to take care of their own needs. *So, the way I was functioning, I’ll tell you, for the … first 3 or 4 months, it was clear, it (my own well-being) took precedence. My priority was more … to take care of myself* (Interviewee A, grandfather). Another had to take care of her mother, who had Alzheimer’s disease, which influenced her grieving process. *I asked myself the other day, … I said to myself, and if Mom had not been there, how would I have gotten through it? It would have been totally different, if you do not have someone to look after 24/7* (Interviewee C, father).

Two participants described the consequences of the suicide on family relationships. At first, there seemed to be some closeness and support. However, for one of the participants, conflicts surrounding the deceased’s inheritance raised tensions, particularly with the extended family. These conflicts over money considerably altered the relationships between family members.

*For the first year, it brought [the family] together. We were really close. As soon as the inheritance of the house came up, then the squabbles erupted. And it went on and on. So now we do not speak anymore. Me, I still say hello, and all that. We had some family dinners where we were really close, and now that’s all finished* (Interviewee A, grandfather). For the other participant, family tensions arose after trying to manage the multiple responsibilities associated with the suicide of their father and the needs of their mother who suffer from Alzheimer’s disease.

*Well, it brought us together because we really had to work together, my brother and me. But I could not do it anymore. I could not stand it anymore. I told myself, after it’s all over, I’ll never speak to him again. Everything was really messed up* (Interviewee C, father).

### Theme 5: Looking for Support

Looking for support was the main coping strategy used by the participants. They all mentioned having received family, marital, or friends’ support. In fact, whether it was the day of the suicide of their close relative or in the short term, they were accompanied by those around them.

*[Interviewer: What helped you deal with this (your grandfather’s suicide)?] The family. At the beginning, we were still quite united. We had family meetings, regularly. Several times, especially the first week, we saw each other all the time. What I think was good anyway* (Interviewee A, grandfather).

On the other hand, we understand from the participants’ discourse that family’s and friends’ help was not always enough and that it is important to receive support from professional or community resources that specialize in services for those bereaved by suicide.

*I think it’s good to consult with someone who is neutral. Because it is good to have family, friends, or other support, but people cannot be completely objective. While an outside person will help mourn the way the person needs to do it […] When I look at families where people do not speak, well if you need help, and someone has committed suicide and you should not talk too much about what you feel, well what do you do with your pain? Where are you going? So, someone neutral, that’s good […] If you do not have it [support], you have to go get it* (Interviewee B, grandaunt).

Even though participants presented a positive perception of the various services they received (social worker, support for Alzheimer caregivers, bereavement counseling center), it appeared however that the bereaved had to do their own research in order to find an organization likely to support them. These specialized services did not come to them automatically.

*But I had to pull myself together so quickly. And then afterwards, I organized myself to get help, I managed to get help from the Alzheimer’s association. At the last minute […] I had started to consult; I had done research I think in December to get help. Because the therapist I had consulted for years, she had retired a few months earlier. And I discovered the Bereavement counseling center. And when I called the line, when I said it was suicide, I was told there was someone specializing in violent deaths who will call me back* (Interviewee C, father). *First step to know: that there are resources, second step, to use them. But still, it’s difficult* (Interviewee A, grandfather).

### Theme 6: The Taboo and Secrecy of Suicide

In general, all participants agreed that suicide is still a taboo subject and that it is an uncomfortable topic for family members and for people in their community. Participant A felt that there is a significant difference between suicide and other types of death: *I think people do not know how to deal with this. It’s not like a death … you know, cancer, or whatever. It’s a suicide. It’s like a taboo* (Interviewee A, grandfather). In fact, the feeling of shame associated with the suicide of a family member is mentioned in the literature as the most common feeling among families, due to the stigma and social prejudice associated with the act. In this sense, it is not surprising that participants’ testimonies revealed that the suicide was surrounded by silence and secrecy.

*What was hard, too, was the fact that it was a suicide. The fact that we know each other. It’s a community, so it’s even smaller, and it was a great burden. […] It’s like you are uncomfortable with everybody. You know that the person knows what happened. You’re not sure how to act with them. You know that it’s really uncomfortable. And everybody else, I think they do not know how to deal with it either […]* (Interviewee A, grandfather).

*We do not talk about … and my mother’s family does not talk about it much. I actually have two families that, … where things are not said. People can think their thoughts, and people would like to have better ties, but we do not talk to each other. So, if someone has suicidal thoughts tomorrow morning, I think that nobody will know about it* (Interviewee B, grandaunt).

The emotionally charged nature of the topic made it difficult to divulge the suicide to others, especially those that are perceived as fragile or vulnerable and believed to be in need of protection (Cerel et al., 2008).

*In the elevator, I decided that I would not tell my mother the truth. With her Alzheimer’s and everything, she would feel guilty. I knew it. I knew her. Emotionally, it’s hard to manage* (Interviewee C, father).

### Theme 7: Perceptions of Aging and the End of Life

Following the suicide of their close older relative, the bereaved participants thought about their own aging. Because they attributed the suicide to age-related loss of faculties, two of the participants expressed fears about ending up alone and useless, losing their autonomy and cognitive abilities, and above all, being placed in a nursing home. Thus, their negative perceptions of aging, which were connected, among others, with the experiences they had gone through with their older relative, seemed to heighten the participants’ fears about getting old.

*I would still like to make some kind of contribution, and not to die alone. With dignity. First, being physically and rationally sound, because you can be one or the other, and it’s no more fun being one more than the other. And yes, if it’s possible, with autonomy. Even if you are not autonomous in every respect, but you are at home, if you have a little help, but you still feel involved. People have to feel useful* (Interviewee B, grandaunt). *Me, I do not want to lose my faculties. You see people, it’s really … at the end, I was at the end of my rope. With two parents, my mother with Alzheimer’s, my father, he was at the beginning … I think that I’m at greater risk than most people. I’m going to write it in my advance directives. I want to make it clear to my daughters* (Interviewee C, father).

Medical assistance in dying (MAiD) was recently legalized in Canada. In the participants’ mind, MAiD offered them an option to avoid problems associated with aging even if this law is accessible only for terminally ill patients. All the participants viewed this as an acceptable way to end a life of unbearable suffering. They felt that the death of their relative would have been less tragic if they had had access to this solution and that the mandatory discussions involved in the MAiD procedure could have resulted at best in a change of mind for their relative.

*I’m glad that we have it now (MAiD), because you can have a degenerative disease, where you know that you are going to die without dignity or forgotten. I mean, you yourself forget things, so do you really want to live like that? Do we have to live like that? Me, I think not* (Interviewee B, grandaunt).

*Because if the person judges that it’s not a good quality of life, like my grandfather said. But instead of doing it the way he did (by suicide), he would have had to get it approved, and people would have had to know about it, talk about it, maybe even get him to change his mind, make him see that he still had some years to live. I think that it (MAiD) would have helped* (Interviewee A, grandfather).

However, participant B had some knowledge about the extent of the administrative procedures attached to the request for MAiD, and she felt that her grandaunt would certainly not be eligible for this life-ending procedure. *I do not know how open she would have been to that, or to fight for that, knowing her. I do not know if she would have been … because it can be long, you know. […] So, I tell myself, would she have fought to demand it? … Take the tests, find out what she had, why she had it. I do not know if she would have done that* (Interviewee B, grandaunt).

## Discussion

The aim of this pilot study was to assess the feasibility of a qualitative research on the psychosocial experience of adults who were bereaved by the suicide of an older person in order to improve the quality and efficiency of the main study. The pilot data presented here are promising and suggest that further investigation is needed on this topic but that some modifications are necessary to improve the methodology (In, 2017), especially on selection criteria and recruitment strategies. As for the semi-structured interview, it covered all pertinent topics and will be suitable for use in the main study. Furthermore, with each interview the main researcher, a doctoral student in psychology, could improve her skills in conducting a qualitative interview, but it was still difficult to seize opportunities for probing emerging topics during the interview process. For example, some participants mentioned seeing their loved one at the scene of the suicide and it would have been relevant to question more about the impact that this had on their grieving process. This will be done in the main study.

Three selection criteria were chosen to ensure that potential participants were not overly vulnerable: age (18 years and over), no depression or suicidal ideation, and bereaved by suicide between 2 and 20 years ago. However, this choice led to a very heterogeneous sample, with only one of the three participants being a first degree relative. We would recommend selecting participants according to the nature of their relationship to the deceased, recruiting separately participants who lost a parent, a grand-parent, or another close elderly relative to suicide. It could also be pertinent to reduce the timespan since the suicide to less than 10 years, but to keep the minimum to 2 years, even if the former would exclude some potential participants. One of the limitations of a retrospective study like ours refers to the fact that participants do not necessarily remember events, that occured a long time ago, in the same way and with equal precision, especially in the case of a traumatic event (Brewin, 2011). Screening for depression and suicidal ideation should be maintained, but participants do not have to be automatically excluded since repercussions on mental health are often consequences of the suicide of a close family member (Figueiredo et al., 2012). By excluding these participants, the portrait of the survivors’ psychosocial experience would be incomplete. However, with a good safety plan, the interview could provide support to participants who do not use mental health services and give them the opportunity to share their experience (Andriessen et al., 2018).

Recruitment difficulties of this pilot study show the challenges that the main research will face to ensure sufficient numbers of respondents. Only three persons accepted to be interviewed; therefore, it was impossible to get enough data to reach saturation, which refers to the point in data collection where new participants do not provide any additional insights (Saunders et al., 2018), and ensure that we have a precise portrait of the psychosocial experience of adults who lost an elderly relative to suicide. Advertisements in public spaces or online by suicide prevention centers were not effective. It could be more appropriate to use individual or professional contacts, referred as the snowball technique, to increase confidence and trust of potential participants and to overcome recruitment problems. In addition, we would suggest reaching out to support groups for those bereaved by suicide even if those participants might present special socio-demographic or motivational characteristics. It may also be relevant to ask the coroner office to send a letter of invitation to participate in the research study to families bereaved by suicide. However, despite the recruitment difficulties, it is important to note that all the participants assessed their experience in the study positively, which corroborates the value of the interviews for those bereaved by suicide (Cooper, 1999; Dyregrov et al., 2011; Andriessen et al., 2018). Thus, this observation supports the feasibility of the study on this aspect. Furthermore, it is important to note that the main study will take place during the COVID-19 pandemic. Thus, the interviews will now be online. We can therefore believe that more people will be able to participate, considering that we will target all regions of the province of Quebec rather than just one. In addition, individuals may be more inclined to participate in the study considering the increase in social isolation associated with the current pandemic (Gunnell et al., 2020).

Qualitative studies of older adults who ended their life are rare as are those that investigate the impact of this event on close family members. Therefore, the preliminary results of this qualitative study fill some gap in the literature on survivors of older adults’ suicide which tended to focus mainly on the spouse (McIntosh, 1993; Clark and Goldney, 2000). The present study explored the effects of the suicide of an older person on other members of the family, in this case, a daughter, a grandson, and a great niece. Interestingly, there were some similarities in the participants’ reports, even if their profiles and the nature of their relationship to the deceased differed in each case. The following discussion will highlight some possible biases that can occur in order to avoid them in the main study.

Results indicated that participants tried to explain and understand the cause of the suicide of their relatives. The search for explanation is a common reaction in all bereaved individuals whether the death is caused by suicide or through natural causes (Harwood et al., 2002). In our study, all participants attributed the suicide to problems associated with aging and that their older relative could not engage anymore in activities that were meaningful to them. As a matter of fact, physical illness, pain, cognitive decline, and fear of placement, as well as poor quality of life, are known risk factors for suicide in older persons (Harwood et al., 2006; Draper et al., 2010; Rurup et al., 2011; Duberstein and Heisel, 2014; Conejero et al., 2018). The various losses faced by the older adults removed opportunities to play key roles in the life of their community or family (whether as caregivers, volunteers, and grandparents) and increased thwarted belongingness, which is a risk factor for suicide (Van Orden et al., 2010). It should be noted that all the participants recruited in this pilot study indicated that their elderly relative suffered from a physical or cognitive decline, even though older adults can intentionally end their life without having a precise reason to do so (Rurup et al., 2011; Van Wijngaarden et al., 2016). Do participants perceive ill-health as an acceptable and understandable reason for the suicide of their loved one and, consequently, are more willing to take part in the research? This result highlights even more the relevance of the coroner’s suicide list as a recruitment strategy to contact various types of survivors to optimize the diversity of the sample.

The participants also felt that suicide was a way for the deceased to regain control over their lives when a disease was threatening their autonomy and quality of life (Rurup et al., 2011). This need for control was also observed in a psychological autopsy study that examined the characteristics of older suicides (Kjølseth et al., 2009). The lack of openness to experience (Duberstein, 1995), the difficulty reengaging in new goals when significant obstacles occur (O’Connor et al., 2009), as well as the feeling of being trapped in an unwanted situation, were also mentioned by participants as personal characteristics of their older relative who died by suicide. The latter supports the psychological theory of suicide of O’Connor and Nock (2014), which considers that feelings of entrapment could be a contributor to suicidal behavior.

It should be noted that two of the older adults had been diagnosed with Alzheimer’s disease. This is not surprising since recent studies indicated that older adults with mild cognitive change and early phase dementia are at increased risk of suicidal behavior, often in the context of comorbid depression (Draper et al., 2010; Conejero et al., 2018). The common belief that dementia is associated with a lack of competence and planning probably increases the shock of the relatives who did not expect their relative to be able to end their life. The main study should expect that part of the sample will have experienced the suicide of an older relative who suffered from cognitive decline and that depressive reactions and suicidal behaviors can be regarded as likely outcomes after the announcement of the dementia diagnosis (Draper et al., 2010).

Seeking reasons for the suicide and reconsidering or restructuring their basic belief system and their world-view appear to help the survivors make some sense of the death (Neimeyer and Sands, 2017). Actually, for many bereaved individuals, suicide is an inexplicable death and they feel the need to understand the motivations and the frame of mind of the deceased (Jordan, 2008). On the other hand, this event also challenged their perception of the world, as a sensible and meaningful place. Suicide can be understood as shattering the assumptive world of the survivor, as destroying the foundational beliefs about their perception of life. As Neimeyer and Harris (2016, p. 165) explain: “a central process of grieving is the attempt to reaffirm or reconstruct a world of meaning that has been challenged by loss”. Participants were clearly changed by the suicide and needed to reconstruct their beliefs about life after it was shattered by the event. Accordingly, some seem to have experienced posttraumatic growth, which refers to positive transformations concerning self-perception, interpersonal relationships, and philosophy of life (Genest et al., 2017). In fact, posttraumatic growth often leads to helping others in a selfless and meaningful way, as evidenced by one of the participants entering the field of gerontology to assist distressed older adults and the fact that all participants wanted to be involved in this study in order to improve knowledge on suicide bereavement. In addition, the nature of the psychiatric disorder of the deceased and its role in contributing to the suicide can be invaluable to survivors. This knowledge can help survivors put their beliefs about their responsibility and the preventability of the suicide into a realistic perspective (Jordan, 2008).

Castelli Dransart (2017) also found that the search for meaning was an important theme in the interviews of 50 survivors, most of them aged below 40 years. Her participants reported four post-suicide challenges: dealing with the impact of the suicide, searching for meaning, clarifying responsibility, and finding a personal style of reaction and coping. These themes validate the results of present study and support the notion that certain ways of reacting to the suicide of a family member are universal, regardless of the deceased’s age.

Furthermore, results indicated that the suicide was a brutal and unexpected event (Figueiredo et al., 2012), even if the participants could have expected the possible death of their loved one due to their health condition. This finding corroborates the results of a qualitative study by Jones (2018) with nine adults bereaved by the suicide of a parent aged 50–65 years. Thus, it appears that the suddenness of the event and the shock at the manner of death contributed to the surreal feelings associated with the suicide, independently of the deceased person’s age. Although the loss of an older relative might be predictable, the manner of death is not.

Participants also expressed anger, especially those who were involved in caregiving and who had spent significant amounts of time seeing to their older relative’s needs. This result is in line with results from Figueiredo et al. (2012), who showed that anger is one of the most common reactions to the suicide of an older relative. The mourners usually considered the suicide as a display of contempt, or as an ungrateful response for the care that was given.

Beyond the impacts on their personal and practical lives, participants also described the consequences for their family. These included tensions between family members and conflicts surrounding the deceased’s inheritance. This concurs with the results by Figueiredo et al. (2012), who noted that instead of joining together in mutual support, family members tend to distance themselves from each other following a suicide, and even more so when financial disputes arise. However, one of the problems inherent to family research on suicide survivors is that the quality of the previous relationships within the family is rarely assessed, making it difficult to comment on the specific implications of the death for family relationships and communications in the aftermath of the suicide (Cerel et al., 2008). Thus, it might be relevant to question the participants during the semi-structured interview not only on the impact of suicide on their family, but also on relationships and family dynamics before the suicide of their older relative.

We also noted that although all the participants agree that it is important to receive support from specialized professionals, they said it was a challenge to find and contact them. Despite the lack of controlled empirical study, clinical experience has highlighted a number of interventions or programs that may be of help to suicide survivors. Thus, despite the various existing resources in postvention (Andriessen and Krysinska, 2012), the study points out to the importance of offering these services systematically on a continuous basis. Indeed, numerous challenges remain with regards to program and policy development, research and clinical practice, to ensure effective care for those bereaved by suicide (Andriessen and Krysinska, 2012).

As in other studies (Harwood et al., 2002; McMenamy et al., 2008; Figueiredo et al., 2012; Jones, 2018), participants expressed guilt and regrets following the suicide of their relative. However, one participant reported no such feelings, considering that she gave a lot of time to her parents. It seems that feelings of self-blame may emerge when grieving survivors continue to ask themselves “Why?” and “What if?” (Capuzzi, 2004). Suicide also had repercussions on the mental health of the participants as observed in several studies on the impact of close family members who intentionally ended their life (Jordan, 2008; Tal Young et al., 2012; Pitman et al., 2014).

In addition, the participants stressed the fact that suicide by an older person is a taboo and secret topic, corroborating the stigma displayed toward suicide and the survivors (Cerel et al., 2008; Sudak et al., 2008). In the case of suicide by an older person, some hypotheses have been proposed to explain this. For instance, the passive and fragile image of older people appears to be inconsistent with the act of suicide, which is viewed as both active and violent. In addition, most religions prohibit suicide. Furthermore, there is little media coverage on suicides by older people (Éthier et al., 2014), as most reported suicides are enacted by younger people. This stems from a social belief that younger people are at greater risk for suicide, which distracts the public from other age groups. Consequently, the general population has little awareness of the fact that other age groups die by suicide, including older persons.

Furthermore, it is possible that the taboo and secrecy surrounding the suicide, in addition to the guilt and regrets felt by the participants, contributed to the recruitment difficulties. Shame may make it exceptionally difficult for family members to speak about the topic of suicide (Cerel et al., 2008). Moreover, even when survivors are not actually avoided by others, they may incorrectly expect to be judged harshly by others. Together, these difficulties may create a cycle of misunderstanding and avoidance with regard to participating in a research on suicide bereavement. Therefore, recruitment difficulties might partly lie in the experience of stigma or shame. Nonetheless, benefits of research participation, reported by traumatized populations, include reducing stigma, normalizing trauma-related reactions, and ensuring safe disclosure of trauma-related information (Newman and Kaloupek, 2004). Publicity about the research should address these beliefs for potential participants and increase their confidence in the compassionate attitude of the researchers as well as the possible benefits of their participation.

One unique component of this study was that participants were asked to reflect about how the recently legalized MAiD would influence the way they see aging and the suicide of their relative. Fear of aging was strong, partly because of the experience they went through with their older relative. However, MAiD seemed to offer them an alternative to dependency and cognitive decline should they eventually consider their lives to be no longer worth living (Van Wijngaarden et al., 2016). The participants’ interest in MAiD could indicate a tendency to consider this practice as a socially acceptable alternative to suicide (Mishara and Weisstub, 2018) and would endorse the position that death is a rational solution to the problems of old age (Yuryev et al., 2010). Therefore, in the context of MAiD, it would be pertinent to add some questions to the interview of the main study about participants’ perception of the help and support that was offered by health care providers to their older relative. What was done to relieve the older adult’s distress, alleviate the suffering, and prevent the suicide? Did they feel that, for the care providers, the life of an older person was deemed worth saving? These perceptions could influence the risk of suicide in survivors when they will face their own difficulties associated with the aging process.

Future studies could also investigate the consequences of suicide on the survivors. For example, evidence suggests that suicide survivors may be at greater risk than other bereaved individuals for a variety of psychological problems, including posttraumatic stress disorder (PTSD) and suicidal behavior (Mitchell et al., 2004; Jordan, 2008; Pompili et al., 2008). It would be useful to add questions on these topics to the semi-structured interview. Furthermore, as proposed by Levi-Belz and Lev-Ari (2018), it is important to examine the personal factors that may help suicide survivors deal with, and recover from, their devastating loss. In fact, their results indicated that securely attached individuals scored highest on posttraumatic growth compared with other attachment styles. Hence, it would be informative to add some questions to address the participants’ attachment style in order to corroborate (or not) Levi-Belz’s and Lev-Ari’s (2018) observation.

## Conclusion

This pilot study investigated the experience of three adults who were bereaved by the suicide of an elderly relative they felt emotionally close to. It showed that like other people bereaved by suicide, those who lost an older family member experienced this event as a shock that significantly perturbed their life. The main difference was related to the fact that the latter found explanations to the suicide in the difficulties associated with the aging process, such as physical and neurocognitive disorders, as well as dependency to others. This perspective seemed to increase participants’ fear of growing old and the need to find a solution to avoid it, such as the recently legalized medical assistance in dying. In conclusion, this pilot study indicates that it is possible to investigate this topic with some modifications to the selection criteria and recruitment strategies for the main study.

## Data Availability Statement

Datasets are available on request to the corresponding author.

## Ethics Statement

The study was reviewed and approved by the Human Research Ethics Board of the University of Québec in Trois-Rivières. The participants provided their written informed consent to participate in this study.

## Author Contributions

GM-D and SL contributed substantially to all research phases, including study conception and design and data analysis and interpretation. GM-D, SL, and CV-Q contributed to data interpretation, manuscript writing and final approval, and critical revision. All authors contributed to the article and approved the submitted version.

## Conflict of Interest

The authors declare that the research was conducted in the absence of any commercial or financial relationships that could be construed as a potential conflict of interest.

## Footnotes

^1^Approved on September 5, 2018 (CER-18-248-07.23).

## Appendix 1: Semi-Structured Interview

Today, we’d like you to talk about your relative and how his/her suicide affected you and the people close to you.

1. Tell me about your relative. What kind of person was he/she? How would you describe your relationship with him/her?
2. Tell me about the day that your relative died by suicide. How did it happen? How did you learn about it?
3. Tell me how you reacted to the loss of your relative to suicide. How did you feel about the act and the reasons why he/she did it?
4. How have you been coping with this?
5. How has your relative’s suicide influenced your life? (your relationships, the way you look at life, and the way you think about your close relative)
6. Have you managed to find hope, comfort, or acceptance since the suicide of your relative?
7. How has the suicide of your relative made you feel? How has it affected the way you function now?
8. Tell me about your family. How did they react to the suicide of your relative? Has this brought you closer to them or created more distance?
9. Has the suicide of your relative changed your relationships with other people in your life, such as friends or colleagues? If yes, in what way?
10. Have there been any other suicides by older people in your family? (If yes, repeat the previous questions for this family member)
11. What suggestions would you give to other people who might be in the same situation?
12. There’s been a lot of talk in the media about medically assisted dying. How does this affect the way you view the suicide of your relative?
13. How do you view old age? How do you feel about growing old yourself?
